# A mixed method investigation of teacher-identified barriers, facilitators and recommendations to implementing daily physical activity in Ontario elementary schools

**DOI:** 10.1186/s12889-022-14359-3

**Published:** 2022-10-31

**Authors:** Lauren Martyn, Hannah Bigelow, Jeffrey D. Graham, Michelle Ogrodnik, Deborah Chiodo, Barbara Fenesi

**Affiliations:** 1 Faculty of Education, Western University, London, ON Canada; 2 Faculty of Social Sciences, Brock University, St. Catherines, ON Canada; 3 Department of Kinesiology, Faculty of Science, McMaster University, Hamilton, ON Canada

**Keywords:** Daily physical activity, Classroom physical activity, Physical activity policy

## Abstract

**Background:**

Fewer than 17% of children worldwide are meeting the international recommendations for daily physical activity. Since most children are in school for the bulk of their day, the classroom has been identified as an ideal space to incorporate physical activity opportunities. In Ontario (Canada), the Daily Physical Activity (DPA) policy aims to ensure all elementary school children receive a minimum of 20 min of moderate to vigorous physical activity each school day during instructional time. However, a 2015 evaluation found that only half of Ontario teachers were meeting this expectation; this work advocated for additional research to monitor implementation and its predictors and to further identify fidelity recommendations. Thus, the current study investigated contemporary factors influencing DPA fidelity in Ontario elementary schools and provides teacher-identified recommendations to support DPA implementation.

**Methods:**

The first part of the study was a quantitative approach surveying 186 elementary school teachers across Ontario. Descriptive statistics including frequencies and means were used to characterize barriers, facilitators, and recommendations to DPA implementation. Spearman’s correlations were used to assess the relation between the likelihood of DPA implementation and intrapersonal factors of gender, teaching experience, prior DPA training and personal physical activity participation. The second part of the study consisted of a qualitative approach using teacher interviews to explore in-depth teachers’ recommendations to support DPA implementation. A thematic analysis was used to analyze the transcripts and identify recommendations for DPA.

**Results:**

Survey results showed that only 23% of teachers met the mandated 20 min of DPA per day. Barriers to implementation included space and time constraints, inadequate training, student behavioural issues and low self-efficacy. Gender, teaching experience and prior DPA training were not related to the likelihood of DPA implementation. Teachers who rated themselves as more physically fit were more likely to implement DPA. Teacher interviews elucidated key areas for improving DPA implementation including greater DPA training opportunities, resources, community partnerships, accountability and strategies that support school-wide implementation.

**Conclusion:**

The current study demonstrated that fidelity to the DPA policy in Ontario elementary schools is on the decline. This work highlights unique factors implicated in DPA fidelity and brings to the forefront teacher recommendations to improve DPA implementation.

## Introduction

Canadian children are experiencing a rise in overweightness, obesity, and comorbid health concerns [1, 2]. The most recent analysis of obesity among children aged 3–19 in Canada found a prevalence rate of 13% [3, 4]. However, the combined prevalence of overweight and obese children aged 2–17 increased from 15 to 26% in Canada between 1978 and 2004 [5]. It is projected that more than one in three Canadian adults will be obese by 2031 if trends continue. There is a dire need for early intervention to transform the trajectory of children’s health and wellbeing. Several factors have been identified as contributors to weight-related health concerns, including genetics, environment, nutrition, and social influences such as sedentariness and physical inactivity [6, 7]. Of these factors, physical inactivity and sedentarism are the most modifiable factors to improve the health of children regardless of socioeconomic status, ethnicity, or genetics [6, 7].

Physical activity can counteract the adverse outcomes of long-term sedentariness which include decreased fitness levels, lower self-esteem reports, and decreased academic achievement [8]. Physical activity is defined by the World Health Organization (WHO) [9] as “any bodily movement produced by skeletal muscles that requires energy expenditure above resting level”. Physical activity can include exercise, but may also include bodily movements such as playing, working, active transportation such as biking, house chores, or recreational activities. Meeting the WHO guidelines of daily physical activity, along with meeting recommended sleep and screen time, can significantly reduce the odds of obesity among children [10]. Furthermore, engaging in long-term, regular physical activity may modify and regulate the structures and functions of the brain that underlie cognition and behaviour [11, 12]. Indeed, physical activity has been shown to improve executive functioning and academic achievement among children [13–16], along with psycho-emotional functioning such as mood, affect, and self-efficacy in diverse populations [17, 18].

Given that most children spend much of their waking hours in school, classrooms provide an ideal space for increasing physical activity while simultaneously decreasing sedentary time. Several systematic reviews have shown that elementary classrooms that are physically active support greater academic achievement compared to traditional sedentary elementary classrooms [14, 19–21]. Children who participate in classroom-based physical activity are also more likely to meet the WHO’s recommendation of 60 min of DPA [22]. Classroom-based physical activity has also been shown to increase students’ feelings of joy and motivation to learn, as well as positive classroom behaviour (i.e., time-on-task) and various aspects of academic achievement; these effects can be seen both acutely and over the long-term [19, 23–25]. Students who are more physically active are also less likely to suffer from mental health concerns such as depression and anxiety [26–28]. Physical activity participation during childhood has also been shown to carry forward into adulthood [8]. It is evident that increasing physical activity in the classroom can support children’s physical and mental health, cognitive functioning, and academic achievement, and can create long-lasting habits into adulthood.

In Canada, several provinces and territories have implemented a Daily Physical Activity (DPA) policy to promote active lifestyles for children in school settings [29]. In Ontario, the DPA policy aims to ensure all elementary school children receive a minimum of 20 min of moderate to vigorous physical activity each school day during instructional time [29]. However, a 2015 evaluation found that only half of Ontario teachers were meeting this expectation [30]; this work advocated for additional research to monitor implementation and its predictors and to further identify fidelity recommendations.

The social ecological model [31, 32] and the social cognitive model [33, 34] have been used in previous research to elucidate barriers and facilitators of DPA. While the social ecological model explains behaviour by examining the dynamic interrelations among various personal and environmental factors influencing behaviour, the social cognitive model suggests than an individual’s knowledge acquisition can be directly related to observing others within the context of social interactions and experiences. Taken together, these models highlight that barriers to and facilitators of DPA can be found within and between teachers, as well as among students, principals, school boards, community values and institutional policy. Specific barriers previously identified have included space and time constraints, lack of administrative support and resources, and a bias towards academic learning [30, 32, 35–41]. Facilitators have been identified as teacher attitude and self-efficacy, student benefits such as increased attention, learning and classroom enjoyment, and institutional support such as available resources and providing gym and outdoor spaces [30, 34, 35, 39, 42]. Previous work has also identified general recommendations for improving DPA implementation such as increased teacher training, greater accountability for monitoring DPA implementation, and integrating DPA across other curricula [30, 38]. However, more specific teacher-identified recommendations are needed to foster actionable change by schools and teachers. In addition, a major tenant in the social cognitive model is the concept of ‘mastery experience’ [43]. This concept refers to experiences that are necessary opportunities that individuals require to hone their skills and develop self-efficacy by successfully completing tasks, and which increases the likelihood of performing a behaviour. It is vital for research to capture what experiences can be offered to teachers to increase their sense of mastery, as this is integral to fostering behaviour change (e.g., increased DPA implementation).

The current study used a sequential mixed-method design to better understand the teacher-identified factors influencing DPA in elementary school classrooms within Ontario, and to identify specific recommendations for improving DPA implementation. The first part of the study was a quantitative approach surveying elementary school teachers from Ontario to (1) further characterize teacher experiences and perceptions of DPA, (2) to determine whether there are specific differences in perspectives or experiences between implementors and non-implementors of DPA, and (3) to determine whether intrapersonal factors not previously examined in the literature (e.g., teacher physical activity participation, gender, teaching experience in years, prior DPA training) influenced the likelihood and extent of DPA implementation. The second part of the study was a qualitative approach using teacher interviews to explore in-depth teachers’ recommendations to support DPA implementation. Importantly, previous work has primarily focused on identifying barriers and facilitators to DPA implementation. However, it is equally important to gauge teachers’ recommendations for DPA fidelity using interviews so that there is greater opportunity to unpack complex themes surrounding implementation.

## Method

### Survey design and participants

To achieve an acceptable margin of error based on a population size of Ontario teachers who teach elementary grades (in 2019–2020 it was 85,538) we recruited a total of 201 participants over a two-month data collection period (May 1 to June 30, 2020). Fifteen participants did not complete all components of the survey and therefore were removed from the dataset (N = 186) leading to a 7% margin of error with 95% confidence intervals. The survey was open to all participants who taught between grades 1–8 in Ontario publicly-funded school boards, and who had at least one full school year of experience in a substitute, long-term occasional or full-time teaching position. Participants were recruited through personal and public social media accounts, as well as through snowball sampling. Digital poster advertisements were shared in the teacher Facebook groups “*Ontario Teachers Resource and Idea Sharing*” and “*Ontario Educators and Mental Health*”. The online poster provided a direct link to the online survey.

The survey consisted of 56 questions and used a mix of multiple-choice, single choice and short answer questions to query participants about their demographic information, their experience and willingness to implement physical activity within their classroom, attitudes towards physical activity in the classroom, and their own personal experience with physical activity. Participants received $10 Canadian for their participation in the form of an Amazon gift card. The study was fully approved by the institution’s research ethics board.

**Survey measurements.*Physical activity in the classroom.*** Questions assessing teachers’ implementation of physical activity within the classroom were based on work by Dinkel et al. [34] who piloted questions with teachers, academic experts, and community health experts. The survey was validated in a previous study measuring the willingness of teachers to implement physical activity and the socio-ecological model [44].

***Attitudes towards physical activity in the classroom.*** An adapted version of the Attitudes Towards Physical Activity (ATPA) questionnaire [45, 46] was used to measure the attitudes, beliefs, and self-efficacy toward physical activity implementation within the classroom. The ATPA was validated in previous work assessing attitudes toward physical activity [45].

***Teacher physical activity behaviour.*** Questions related to aerobic physical activity and perceived aerobic fitness from the Canadian Society for Exercise Physiology (CSEP) Physical Activity and Sedentary Behaviour Questionnaire (PASB-Q) were used to measure teachers’ personal physical activity behaviour [47]. The PASB-Q was validated in previous work assessing physical activity and sedentary behaviours among adults [48].

### Interview design and participants

Interviews were used to elucidate teacher-identified recommendations to support DPA implementation. The recruitment letter used in survey contained a link to sign up for audio-recorded teacher interviews. Fifteen teachers expressed interest in participating. Interviews were conducted with one to three participants based on participant availability. A total of eight groups of interviews were conducted. The interview was semi-structured and conducted in an open-ended manner allowing for participants to engage in a conversation-style interaction. Each interview lasted approximately 1.5 h, and participants were compensated $30 via Amazon e-gift card.

The interview content was based on previous research using semi-structured interviews [34]. Four questions explored participants’ perspectives on how implementation could be improved through questions such as, *“If we were to create training on incorporating physical activity breaks into classrooms, what do you think would be most important to include?”.*

### Statistical analyses

#### Survey analyses

The IBM SPSS statistics software platform (Version 26) was used to carry out all analyses. Descriptive statistics (means and standard deviations for continuous variables, and frequency counts and percentages for categorical variables) were computed to describe demographic characteristics (Table 1) and physical activity behaviours within the classroom (Table 2). The sample size for frequencies in Table 2 varies as some questions allowed for multiple responses. Question 1 in Table 2 was used to dichotomize implementors (n = 159) versus non-implementors (n = 27). Welch’s *t*-tests were used to assess differences in beliefs and behaviours between implementors versus non-implementors of DPA (see Table 3). Correlational analyses were used to assess the relation between intrapersonal factors and the likelihood of DPA implementation. Outliers were removed according to SPSS step of 1.5 x IQR (interquartile range).

#### Interview analysis

Upon completion of the interviews, audio recordings were transcribed using Trint, a cloud-based audio and video transcription tool, to produce written verbatim transcriptions. Transcripts were checked against the recordings to ensure accuracy. A preliminary codebook was developed between the two central researchers based upon the theoretical construct of mastery experience, a key theme in social cognitive theory [43]. The preliminary codebook was then applied to eight interview transcripts. The researchers met and discussed each excerpt they applied to a theme. Any discrepancies were discussed to determine the final decision, such as adding, removing, or redefining codes. Updates to the codebook were applied, and each transcript was re-reviewed with the new criteria.

A thematic analysis was used to analyze the transcripts and identify recommendations for DPA. The thematic analysis was conducted based on six phases summarized by Maguire and Delahunt [49] to increase trustworthiness and rigour. The first phase aimed to familiarize researchers with the data and was accomplished by reading the transcripts to immerse in the data. Two complete reviews of the data set occurred in this phase. The second phase initiated the formulation of preliminary codes. All transcriptions were inputted into Dedoose (V.8.1.8), a qualitative computer software program. In phase three, significant or interesting patterns in the data were combined to make themes and sub-themes. Extracts from the data were then organized under matching themes. The third complete review of the data set occurred after this phase. In phase four, themes were re-reviewed and polished. Any modifications during the fourth phase prompted a re-review of all the transcripts. In phase five, the final refinement of themes was completed by generating a name and definition for each theme. Once all themes were defined, a final review of the data set occurred. Lastly, in phase six, concise, coherent, and interesting excerpts from participants were provided to represent predominant and distinctive themes.

#### Trustworthiness

Several measures were used to consider credibility, transferability, and dependability to ensure the trustworthiness of the research. Both investigator and theory triangulation were employed to ensure credible data [40]. Data triangulation was used in the review of previous research findings with consideration of different time points, location of origin and research methods [50]. In reviewing diverse data, past findings and methods were evaluated to determine consistency with the present study. Investigator triangulation was used throughout the study, with particular focus during data analysis and discussion of findings to minimize researcher bias. Lastly, theory triangulation was used through the implementation of the social-cognitive and social-ecological model to develop research and interview questions, and to guide data analysis [50]. To promote transferability and transparency, the attributes of the study, such as decision-making and justification, were noted in detail through a reflective journal.

## Results

### Survey results

The following tables summarize survey demographics (Table 1), physical activity behaviours in classrooms (Table 2) and differences between DPA implementors versus non-implementors (Table 3).

**Table 1:** Survey demographics

	Variables	N (%)
	Total	186 (100)
Demographic characteristics		
Gender	Man	14 (7.5)
	Woman	170 (91.4)
	Non-binary	1 (0.55)
	Prefer not to answer	1 (0.55)
Age		
	23–29	59 (31.7)
	30–45	98 (52.7)
	46–65	29 (15.6)
	65+	0
Ethnicity		
	Caucasian	156 (83.4)
	Asian	19 (10.8)
	Black	2 (1.1)
	Hispanic	1 (0.5)
	Indigenous	1 (0.5)
	Multi-racial	1 (0.5)
	West Indian (Caribbean)	1 (0.5)
	Prefer not to answer	5 (2.7)
Highest level of education		
	Bachelor’s Degree	144
	Master’s Degree	42
Teaching status		
	Full-time	147 (79.0)
	Part-time	3 (1.6)
	Long-term occasional	6 (3.2)
	Supply	30 (16.1)
Teaching role		
	Homeroom teacher	138 (74.2)
	Supply teacher	13 (7)
	Science	4 (2.2)
	Other	31 (16.7)

*Note.* Teaching role “other” category consisted of math, English, music, learning resource, French, English as Alternate Language, art, dance, drama; all “other” categories consisted of < 2% of sample and therefore were not individually listed

**Table 2:** Physical activity behaviours in the classroom

Survey Questions	Responses	Frequencies (%)
1. Do you incorporate DPA into your classroom?		N = 186
	Yes	159 (86)
	No	27 (14)
Teachers who implement DPA		
2.What courses are you most comfortable implementing DPA within?		N = 217
	All courses	71 (32.7)
	Language	39 (18)
	Math	37 (17.1)
	Transitions between lessons	11 (5.1)
	Science	10 (4.6)
	Other	49 (22.5)
3. How many days/week do you incorporate DPA?		N = 159
	1–2 days/week	4 (2.5)
	2–3 days/week	26 (16.4)
	3–4 days/week	41 (25.8)
	4–5 days/week	25 (15.7)
	5 days/week	56 (35.2)
	Prefer not to answer	4 (2.5)
	When no physical education class	3 (1.9)
4. How many times/day do you incorporate DPA?		N = 159
	1–2 times/day	107 (67.3)
	2–3 times/day	34 (21.4)
	3–4 times/day	8 (5.1)
	4–5 times/day	5 (3.1)
	Prefer not to answer	5 (3.1)
5. How many minutes is each DPA bout?		N = 159
	1–5 min	57 (35.8)
	5–10 min	49 (30.8)
	10–15 min	31 (19.5)
	Over 15 min	22 (13.8)
6. What is the most common type of DPA you used in your classroom?		N = 377
	Dancing	71 (18.8)
	Movement videos	69 (18.3)
	Yoga	42 (11.1)
	Stretching	41 (10.9)
	Cardio	36 (9.6)
	General movement	35 (9.3)
	Games	35 (9.3)
	Walks	20 (5.3)
	Outdoor play	15 (4.0)
	Other	13 (3.4)
7. Does DPA include an academic component?		N = 159
	Yes	126 (79.2)
	No	33 (20.8)
8. What are the most observed benefits of DPA?		N = 288
	Increases focus and learning	102 (34.2)
	Brain break	36 (12.1)
	Improves health	25 (8.4)
	Releases energy	25 (8.4)
	Activates body	24 (8.1)
	Energizes student and teacher	20 (6.9)
	Increases peer engagement	14 (4.7)
	Increases relaxation	12 (4.0)
	Other	30 (10.4)
9. What are some barriers to incorporating DPA?		N = 245
	Lack of space	103 (42.0)
	Lack of time	71 (29.0)
	Lack of knowledge/resources	14 (5.7)
	Unwilling students	11 (4.5)
	Behavioural issues	11 (4.5
	Other	35 (14.3)
10. How do students typically respond to DPA?		N = 179
	Enjoy	137 (76.5)
	Mixed feelings	12 (6.7)
	Other	30 (16.8)
11. Have you ever received DPA training?		N = 159
	No	93 (58.5)
	Yes – Teachers college	25 (15.7)
	Yes – DPA workshops	24 (15.1)
	Yes - OPHEA	3 (1.9)
	Yes – Peers	1 (0.6)
	Prefer not to answer	13 (8.2)
Teachers who do not implement DPA		
12. Are there any reasons why you do not incorporate DPA into your classroom?		N = 37
	Lack of time	14 (37.8)
	No training	7 (18.9)
	Lack of space	5 (13.5)
	Other	11 (29.7)
13. Would you consider incorporating DPA?		N = 27
	Yes	22 (81.5)
	No	1 (3.7)
	Unsure	4 (14.8)
14. What knowledge or resources would help facilitate your implementation of DPA?		N = 38
	More access to resources	9 (23.7)
	More training	8 (21.1)
	How to incorporate DPA into lessons	5 (13.2)
	Prefer not to answer	9 (23.7)
	Other	7 (18.4)

*Note.* All “other” responses include frequencies of < 10; Q1 “other” includes drama, music, art, social studies, dance, health, history, end of day, religion and morning and end of day. Q6 “other” includes follow the leader and prefer not to answer. Q8 “other” includes models healthy living, breaks up lesson, increases physical engagement with learning, increases academic enjoyment, helps transitions and prefer not to answer. Q9 “other” includes inclusion, lack of training, self-conscious students, student buy-in, lack of institutional support, unwilling teacher, discomfort leading physical activity, exhausted teacher, prefer not to answer. Q10 “other” includes look forward to it, embarrassed, request more, view as distraction, unwilling, need to be motivated, don’t enjoy, prefer not to answer. Q12 “other” includes students receive physical activity elsewhere, academics more important, unwilling students, disruptive, teach special education, personally don’t enjoy physical activity. Q14 “other” includes knowledge on how to make DPA accessible and how to maximize use of space, more time, more space, more institutional support

Using questions 3–5, a composite score was created to represent the average number of minutes per week that teachers implemented DPA. One extreme outlier was removed (N = 159 changed to N = 158), with the resulting mean of 64.5 min per week and standard deviation of 53.2 min per week. Similar to previous work that used a DPA fidelity score [30], in order to meet the mandated 20 min per day of DPA, a minimum weekly DPA cut-off was set to 100 min per week. Only 37 of 158 participants met the 100 min per week cut-off (23%).

**Table 3:** Differences between implementors and non-implementors of DPA

	Implementors of DPA N = 159	Non-Implementors of DPA N = 27
	Mean (SD)	Mean (SD)
Characteristics		
Confidence and motivation implementing DPA		
I feel confident implementing DPA.	4.01 (0.8)	3.15 (1.2)**
I can successfully encourage student participation in DPA.	4.01 (0.7)	3.46 (1)*
I feel motivated to incorporate DPA into my classroom.	3.92 (0.8)	2.9 (0.9)**
I feel knowledgeable about DPA strategies and techniques for use in my classroom.	3.49 (1)	2.9 (1.1)**
I feel unsure about implementing DPA.	2.2 (0.8)	3.2 (1)**
Institutional support		
My school promotes messages of health and wellness for students.	3.9 (0.9)	3.7 (1.1)
My school administration supports the use of DPA in the classroom.	3.7 (0.8)	3.6 (0.8)
Other teachers within my school agree with my opinions on incorporating DPA.	3.5 (0.8)	3.5 (0.7)
Other teachers within my district agree with my opinions on incorporating DPA.	3.6 (0.7)	3.3 (0.7)
Classroom characteristics		
There is time in the school day available for incorporating DPA.	3.1 (1.1)	2.5 (1.1)*
When I have incorporated DPA, my students often misbehaved.	2.3 (0.7)	3.1 (1)**
It is feasible to implement DPA into my classroom.	3.7 (0.8)	2.6 (0.9)**

*Note.* * denotes *p* < .05; ** denotes *p* < .01

Pearson correlational analyses were used to assess how intrapersonal factors, such as gender, teaching experience (years), prior DPA training, and individual physical activity participation influenced the likelihood and extent of DPA implementation. Gender and teaching experience did not correlate with the likelihood of implementing DPA (*r*s < 0.12, *p*s > 0.12). Individual physical activity participation correlated with the likelihood of DPA implementation depending on the measure used; when using total minutes per week of aerobic physical activity that teachers personally engaged in there was no correlation with the likelihood of DPA implementation (*r* = .01, *p* = .92). When using self-rated physical fitness, there was a positive correlation such that the higher someone rated their physical fitness, the more likely they were to implement DPA (*r* = .17, *p* = .02).

Among implementors of DPA, gender, teaching experience, prior DPA training and individual physical activity participation did not correlate with the extent to which DPA was incorporated into the classroom (mins/week) (*r*s < 0.15, *p*s > 0.07).

### Interview results

Teacher interviews aimed to elucidate recommendations for implementing DPA. Participant demographic information is provided in Table 4 with pseudnyms to protect their identities. Themes and subthemes are represented in Table 5 in order of highest frequency which was defined by the number of times the theme was mentioned across all interviews. Quotations from participants are provided to support the contextualization of the themes discussed. Findings and quotations reflect the predominant and unique themes identified across all participants. A total of 106 excerpts were categorized across interviews.

**Table 4:** Interview demographics

Name	Gender	Age	Teaching Experience (years)
TR	Female	24	1.5
AN	Female	27	5
BH	Female	28	5
SP	Female	49	25
EY	Male	54	20
CM	Female	39	15
KG	Female	55	30
DF	Female	47	17
OW	Male	44	16
PJ	Female	25	2
MA	Female	27	4
LT	Female	40	2
JB	Male	N/A	N/A

**Table 5:** Frequency of teacher-identified recommendations for DPA implementation

Theme and Subtheme		Frequencies
Training		46
	Implementation Content Diverse/realistic activities Evidence of benefits Minimal space Behavioural management	33 17 10 5 1
	Professional Development	7
	Preservice Training	6
Resources		37
	Resource bank	21
	Easy activities	9
	More space	7
Community Partnerships		10
Policy		8
	Administration accountability	4
	Teacher accountability	4
School-wide DPA		5

#### Theme 1: training (frequency 46)

Participants described recommendations for training to improve their ability to implement DPA. Within training, the three prevalent subthemes were: implementation content, professional development, and preservice training. The subthemes are discussed below.

**Subtheme 1: Implementation Content (Frequency 33).** Participants suggested teacher-training opportunities with targeted content to improve their implementation of DPA. This subtheme included a variety of ideas, such as training on activities that are diverse, realistic and that can be done with minimal space. Training for behaviour management was also recommended; many teachers expressed struggling with DPA implementation as it evoked negative behaviours and derailed classroom instruction. Further, teachers suggested making explicit how DPA benefits students’ physical and cognitive wellbeing as well as their academic performance. As described by one participant:

*In terms of resources, a lot of them don’t translate into what most classes look like today. Seeing real-life examples of what doesn’t necessarily work and how to do it in your class, but better, I think would be beneficial* (TR).

**Subtheme 2: Professional Development (Frequency 7).** Participants suggested professional development for staff to advance their DPA implementation. This theme included providing training for teachers or administrators through professional development days or workshops. This would provide space to share implementation content and ideally opportunities to observe DPA examples through modelling. As noted by one participant:

*An opportunity to try activities out yourself, like a trial or during professional development days where you’re expected to teach a mini-lesson and get teachers familiar with it or to observe other teachers that feel super competent and comfortable teaching DPA. Modelling is helpful, especially when you’re trying something you’re uncomfortable with* (OW).

Another participant, who had some previous training expressed it would helpful to have additional training that showcased different types of DPA activities.

*I learned some simple games, but that was a year ago. So, it’d be nice to have a refresher on that, and [learn additional] simple things that you could do in the classroom* (LT).

**Subtheme 3: Preservice Training (Frequency 6).** Participants suggested training for preservice teachers to support DPA implementation. This theme included DPA training for preservice teachers in teacher education programs. A lack of preservice programming around DPA implementation was consistently noted as a barrier, and many participants voiced that preservice education should devote curriculum to sharing DPA resources and information.

*I think it should be teacher education programs who take the brunt of that because I didn’t see any of that when I went through…In those five years, we didn’t have anything devoted to DPA except for our Phys Ed class, which was one semester. And again, it was kind of large games. If you’re running events at your school, it could be helpful, but not really focused on DPA* (TR).

Another participant expressed empathy for new teachers who enter the role with minimal knowledge and support on implementing DPA, “*For a first-year teacher coming in, I always feel for them because I they come in with nothing and nobody to guide them. Just one extra piece to add to their stress level”* (CM).

#### Theme 2: resources (frequency 37)

Participants described recommendations for resources to improve their ability to implement DPA. Within the theme of resources, the three most prevalent subthemes were: resource bank, easy activities, and more space. The subthemes are discussed below.

**Subtheme 1: Resource Bank (Frequency 21).** Participants suggested a resource bank to support DPA implementation. This theme included ideas of an online, comprehensive, and exhaustive portal of compiled activities. Participants acknowledged that although they did have access to online resources, they were dispersed across many sources making it challenging to select an activity efficiently and spontaneously during instructional time. One participant suggested, “*Perhaps a bank of core ideas and I know there are some that exist out there. But having a full list of indoor activities and outdoor activities”* (MA). Another participant emphasized the utility of a resource bank with a collection of ideas.

*As a teacher, I want variety. Show me new all the time. I get tired sometimes doing the same thing over and over again. It’s nice to get those resources on new, interesting things* (PJ).

**Subtheme 2: Easy Activities (Frequency 9).** Participants suggested easy-to-implement activities to support DPA implementation. This subtheme included ideas for activities that are easy to learn and simple to use; *“Having easy options so it’s not something that you have to think about in your planning because you’re always trying to plan for other things”* (LT). This reflects the previous subtheme and teachers’ desire to have quick access to easily implementable activities through a centralized resource bank.

#### Theme 3: community partnerships (frequency 10)

Participants suggested community partnerships to support DPA implementation. This theme included ideas such as community partners, organizations, and role models. Participants described that community partnerships could help provide students with diverse DPA opportunities beyond the traditional classroom approach while offloading some of workload from teachers. One participant noted:

*There’s a big push to get community members in the school, like elders and so on. But it’s more so focused on passing traditional knowledge. I’ve never seen it incorporated in a fitness-related way. But I can see the benefits of that* (OW).

Another participant suggested high school and elementary schools could collaborate on DPA and discuss their ideas of introducing new activities to students in this proposed community partnership.

*I think there could be better partnerships. I think that might help teachers do more things. Even having some of the high school Physical Education classes [come into the elementary setting] and teach; that would be a great way for them to teach something like football, flag football, or rugby, by the [actual] team. Teaching kids some of the sports that we don’t necessarily see* (SP).

#### Theme 4: policy (frequency 8)

Participants recommended policy changes to improve their ability to implement DPA. The two most prevalent subthemes were administrative accountability and teacher accountability. The subthemes are discussed below.

**Subtheme 1: Administrative Accountability (Frequency 4).** Participants suggested

policies to hold administrative staff responsible for ensuring DPA is implemented among teachers. Lack of oversight from administrators was noted by several participants as a significant factor in what they viewed as poor implementation fidelity. One participant suggested:

*I don’t know how helpful it would be or how realistic it would be for admin to be dropping by, but just something so you’re thinking about it weekly. And if you have that little reminder that I need to report to someone what I did each day. I think something like that is minimal but could be helpful* (OW).

**Subtheme 2: Teacher Accountability (Frequency 4).** Participants suggested policies to hold teachers responsible for DPA implementation. This suggestion was complementary to administrator accountability, and acknowledges that all parts of the school system need to work together to support successful DPA implementation. One participant suggested, “*I think it could be as simple as just sending your admin a quick email weekly of what you did each day just to hold yourself accountable”* (PJ).

#### Theme 5: school-wide DPA (frequency 5)

The theme of school-wide DPA was chosen for its noteworthiness as a theme not previously identified in literature. This theme included ideas to reinstate a previously existing school-wide initiative or to create a new incentive to engage the entire school simultaneously in physical activity. Again, this acknowledges the school as an ecosystem working together to support DPA. One participant described “*Incorporating a daily school-wide [activity], like when we used to do it in the morning, I mean, it was only 5 minutes, but at least it was something and everybody was doing it”* (CM). When asked if a school-wide shared DPA activity would motivate them, another participant responded, “*If everybody was doing it, I would be a 100% sure, let’s do that. If we all started our day at nine o’clock and went outside [to meet the kids] and did something together, that would be great”* (SP).

## Discussion

The current study used a sequential mixed-method approach to better understand the teacher-identified factors influencing DPA in elementary school classrooms within Ontario. Survey results showed while most teachers do implement DPA, the majority fail to meet the recommended 20 min per day. Recurring barriers of available space and time and inadequate training were identified across all teachers, and specific barriers related to lower ratings of self-efficacy were identified among teachers who did not implement DPA. Intrapersonal factors of gender, physical activity engagement, experience teaching, and prior DPA training did not relate to the likelihood of DPA implementation. Promisingly, teachers noted improved attention and learning following DPA and were open to greater implementation if provided with more resources and training. Teacher interviews highlighted several key recommendations for DPA implementation including greater training opportunities, resources, community partnerships, accountability and strategies that support school-wide implementation. The following will discuss the survey and interview results in relation to existing literature, novel contributions, and study limitations.

Survey results demonstrated that most teachers incorporated some form of DPA into their classroom (87%) and that they were comfortable implementing DPA in most courses with language and math specifically noted. However, the average weekly implementation of DPA was only 64.5 min per week, which falls well below the recommended 100 min per week (20 min per day). In fact, only 23% of teachers met the mandated 100 min per week. This is markedly lower than the 50% found in prior work by Allison et al. [30] who had a similar sample size of 209 surveyed Ontario teachers. The lower percentage of DPA fidelity in the current study raises concerns over whether DPA implementation is further declining in Ontario elementary schools. Most teachers indicated that their DPA includes an academic component (79%) which suggests most classes are learning while engaging in physical activity. The most common activities included movement videos and dancing, and the most common duration implemented was between 1 and 5 min. Teachers indicated the most observed benefits of DPA were increased focus and learning, and 76.5% of teachers believed that their students enjoyed DPA. This finding aligns with prior work indicating students enjoy classroom DPA [38, 41, 51]. The most common barriers identified were lack of time and available space, which also supports previous findings [30, 32, 35–38, 40, 41]. Additionally, despite previous recommendations to the Ontario Ministry of Education [30] that teachers receive more DPA training, the current work found that more than half of teachers still received no DPA training (58.5%). Most teachers who reported not implementing DPA indicated that they would consider implementation (81.5%) if provided with greater access to resources, knowledge, and training around DPA implementation. Interestingly, both implementors and non-implementors indicated that their peers supported their views on DPA implementation. This may suggest that those who implement DPA mainly discuss classroom physical activity with others who implement DPA, and those who do not implement DPA mainly discuss classroom physical activity with others who do not implement DPA. For non-implementors of DPA, this could potentially create a feedback loop where one’s teaching approaches are reinforced rather than challenged and diversified. This finding further underscores the importance of training opportunities where evidence-based perspectives on the benefits of DPA can be shared, while also creating a safe space to voice differing opinions on DPA. It is evident that there are many challenges associated with DPA implementation; ensuring everyone can voice their concerns while simultaneously receiving implementation support is an important part of the fidelity-promoting process.

Survey results also yielded several key distinctions between those who implemented DPA and those who did not implement DPA. Teachers who did not implement DPA had lower ratings of confidence and motivation for implementation, rated the implementation of DPA less feasible in their classroom, rated themselves as having less time in the day for DPA, and noted a higher incidence of student disruption during DPA. These results align with previous research showing that teacher self-efficacy is an important component of DPA implementation [30, 42, 52, 53], and that notable barriers include time constraints and student behavioural issues during DPA [30, 32, 35–41]. Intrapersonal factors of gender, teaching experience (years), personal physical activity behaviour and prior DPA training did not relate to the likelihood of DPA implementation. Prior work suggested that years of teaching experience may be a key factor in the likelihood of teachers implementing DPA [34]; however, this work suggests that teaching experience did not relate to the likelihood of implementation. Furthermore, while previous work has suggested that teachers’ own physical activity and wellness experiences may impact their desire to implement DPA [34, 54, 55], the current study found no correlation between personal physical activity behaviour and likelihood of DPA implementation. The only intrapersonal factor that correlated with the likelihood of DPA implementation was self-rated physical fitness, such that teachers who rated themselves as being more physically fit were more likely to implement DPA. This finding supports previous interviews with teachers who stated that they believed colleagues who did not implement DPA had differing philosophical perspectives surrounding the benefit of DPA for classroom learning [34]. This finding also suggests that psychological perceptions of physical fitness may be as important as actual physical activity engagement when it comes to the likelihood of implementing DPA.

The barriers identified throughout the survey results are in alignment with both social ecological and social cognitive models. From a social ecological perspective, the barriers reflect a combination of intrapersonal, interpersonal, and institutional level factors impacting DPA implementation [30–32, 56]. Importantly, while institutional factors were rated as impacting DPA implementation when all teachers were considered together, when considering implementors versus non-implementors separately, institutional factors was not as significant of a driving force in their DPA differences. Rather, greater emphasis was on intrapersonal, interpersonal, and classroom-level factors. This finding aligns closely with work by Allison et al. [59] who concluded that teacher-level and classroom-level factors were the most likely to impact DPA fidelity. These results could be viewed as a positive finding given that factors closer to an individual’s influence (intrapersonal, classroom) may be easier to modify to exact behaviour change [31–35, 56, 57]. From a social cognitive perspective, self-efficacy was a major component in DPA implementation, such that teachers who had higher ratings of self-efficacy were more likely to implement DPA [33, 34]. Identification was another component important to DPA implementation, such that teachers who identified as more physically fit were more likely to implement DPA. This is also related to social ecological intrapersonal factors and emphasizes the importance of creating a broader culture that values physical activity and its influence on overall wellbeing, including cognitive and academic achievement [58–60].

Interview results elucidated important areas for supporting DPA implementation. Teachers requested a combination of DPA training opportunities and access to diverse and realistic activities. This reflects specific barriers identified in the survey results and in prior research, such as the need for accessible resources that target diverse ages, cultures, and physical space restrictions [30, 32, 34, 35, 40]. Although there are some resources available to teachers to support DPA implementation (e.g., OPHEA), the awareness of these resources may be the key limiting factor. Better connecting teachers with available resources through professional development opportunities, or during preservice programming, may help support their ability to implement DPA. Insufficient training has appeared numerous times in previous research as a significant barrier to DPA [30, 32, 34, 35, 40] and this study underscores how teachers recognize this as a significant area of need for their professional development. From a social cognitive theory perspective, the desire for mastery experiences with a knowledgeable model offers a step towards increasing self-efficacy [33], which is an essential intrapersonal factor to promote DPA fidelity [30, 34, 42, 52, 53].

Teachers also noted that training opportunities should emphasize the evidence-based benefits of DPA for student learning and wellbeing. Useful information could include how children who engage in classroom DPA are more likely to meet the WHO’s recommendation of 60 min of daily physical activity [22], are more likely to perform better in school [19–21, 23, 24], are more likely to be joyful and motivated to learn [19] and are more likely to spend time on task in the classroom [61]. This suggestion is related to previously identified barriers involving the devaluing of physical activity at intrapersonal and school levels [32, 35, 40]. Both social cognitive and social ecological theories agree that behaviour modification requires a shift in beliefs at both personal and systemic levels. Interestingly, recent work by Allison et al. [56] aimed to identify the extent to which school (administrative) factors versus teacher and classroom factors influenced DPA implementation and found that the most effective way to address DPA fidelity was to target teacher and classroom factors; these factors included teachers’ DPA perceptions, teachers’ self-efficacy, classroom DPA scheduling, and issues surrounding lack of space and time. The current study echoes these findings by further emphasizing the particularly important role that teachers’ perceptions and their self-efficacy, along with classroom barriers such as lack of space and time, play in the success of DPA. Together these studies suggest that implementation fidelity may be better supported by focusing less on school/administrator predictors of DPA, and focusing more on teacher and classroom predictors of DPA. Furthermore, infusing DPA training with the science behind the benefits of classroom-based physical activity for cognitive and psycho-emotional functioning is a viable catalyst to encourage greater teacher involvement in DPA. Additionally, finding solutions for the recurring theme of lack of space is imperative [30, 34, 56]. Teachers suggested creating designated DPA spaces, such as unused classrooms. Although not all schools may be able to support this suggestion, it highlights how teachers are expected to implement DPA but feel that there is a lack of available space.

Teachers also suggested support from the community to help teachers implement DPA through volunteer, elders, sports organizations, and community activity initiatives. Community involvement would not only help ease teachers’ DPA demands, but it could also directly help community partners. For example, sports organizations that run a DPA example of their sport may benefit from an increase in sign up by students. High school students who need volunteer hours could also organize a class’s DPA to benefit the teachers, older students, as well as bring new excitement to the class. Work by Tremblay and colleagues [8] similarly suggested integrating community-based resources with preschool education to promote DPA. The current work elucidates a similar recognition of the benefit of community partnerships to support DPA at the elementary level. Importantly, the integration of community may enhance social norms around the value of DPA as well as provide teachers with opportunities for observational learning and modelling DPA positive behaviours [30, 32, 34, 35, 40, 56].

Teachers recommended policy revitalization to foster implementation of DPA through enhanced administration and teacher responsibility. Currently, the mandate does not specify who is directly responsible for ensuring students achieve their 20 min of DPA [62]. It may be helpful for roles at various staff levels to be clearly defined in a revised policy. Having different staff responsible for implementing, enforcing, and ensuring adequate resources are available could increase and distribute accountability. While teachers only specified policy changes related to accountability, many of the key areas identified as recommendations could embed policy. The importance of accountability in DPA fidelity has been identified as a central factor in several prior studies [8, 22, 30, 58, 63, 64]. Furthermore, the current study identified policies for key areas such as teacher education, training, and more specific guidelines, as well as the integration of community-based resources and services.

Additionally, teachers recommended initiating or reinstating school-wide DPA engagement. Some teachers reported that their school once had some form of school-wide implementation, such as an activity over the morning announcements in which the whole school participated. These teachers found it helpful for time management as DPA was always at a scheduled time and fostered a positive DPA school culture. Reasons as to why the school-wide DPA was removed from some schools remains unclear. Further, teachers who did not have experience with a school-wide DPA activity endorsed this initiative, citing it would help alleviate their stress with meeting academic demands by having one less responsibility. This recommendation is supported by prior work arguing that addressing school-level barriers to DPA is integral to promoting classroom implementation [30, 32, 34, 35, 40, 56].

Similar to the survey results, elements of both the social cognitive theory and social ecological model were found throughout the interview outcomes, with the most distinct findings around social ecological model levels such as intrapersonal, interpersonal, institutional and community [30, 35, 56–60]. Teachers’ recommendations targeted their social support and connection among different levels of their social environment, as well as their self-efficacy through training and modelling opportunities [30, 34, 56, 57]. Components of the social cognitive theory that were most frequently identified included self-efficacy, behavioural capability, expectations, and observational learning [32, 34]. Interviews offered an in-depth perspective on DPA recommendations directly from teachers and is a vital step towards identifying ways to better support DPA fidelity. An important area for future investigation is examining how pre-service teacher education can support DPA training even before teachers enter the classroom.

## Limitations

There are several limitations to consider. First, the sample size for both the survey and teacher interviews could have been larger to better represent the population of teachers within Ontario. However, the survey sample did achieve an acceptable margin of error at 7% and although the interview sample size was small, it did meet the principle of saturation and provided new and resonant information [65, 66]. Second, convenience sampling limits confidence in the sampling pool being accurately representative of Ontario teachers. However, due to the COVID-19 pandemic, previous connections to school boards were not accessible and therefore recruitment was done through channels of personal connection or knowledge. While teachers were asked to reflect on their DPA practices prior to the pandemic and thus the current data reflect traditional classroom practices, it would be valuable to revisit how the demands of teaching in a COVID context has further altered DPA fidelity. Additionally, given that some interviews were group-based settings, and some were individual settings, this may have introduced variability in participant comfort levels when sharing their views. These interview structures were selected based on scheduling limitations during the height of the COVID-19 pandemic as it was challenging to organize mutually agreed upon times. Lastly, future work should explicitly ask teacher participants whether they are aware of DPA policies, as this was missing from the current study’s analysis. This would provide critical insight into whether limited DPA fidelity is first and foremost based on a potential lack of information.

## Conclusion

The current study demonstrated that fidelity to the DPA policy in Ontario elementary schools is on the decline. Although most teachers do implement some amount of DPA, they need more support to meet the DPA requirements of 20 min per day. These supports include greater DPA training through pre-service education or professional development to help increase self-efficacy and troubleshoot classroom issues, greater accountability, and access to DPA resources, and improved school-wide initiative. Creative solutions involving community partnerships were also identified to support DPA implementation. Taken all together, this work underscores the importance of working with teachers and educators to identify areas for improving physical activity participation during the school day. Teachers and classrooms play a pivotal role in increasing children’s physical activity levels, which is fundamental for their physical and mental health across their lifespan.

## Data Availability

The datasets analysed during the current study are available from the corresponding author on reasonable request.
